# Successful immunotherapy with ipilimumab and nivolumab in a patient with pulmonary sclerosing pneumocytoma

**DOI:** 10.1007/s13691-024-00737-8

**Published:** 2024-11-29

**Authors:** Yumi Inukai-Motokura, Kiichiro Ninomiya, Takahiro Baba, Hiroki Omori, Tetsuya Takeguchi, Mari Uno, Yoshiyuki Ayada, Takehiro Tanaka, Yoshinobu Maeda, Kadoaki Ohashi

**Affiliations:** 1 Department of Allergy and Respiratory Medicine, Okayama University Hospital, Okayama, Japan; 2 Center for Comprehensive Genomic Medicine, Okayama University Hospital, 2-5-1 Shikata-cho Kita-ku, Okayama, 700-8558 Japan; 3 Department of Pathology, Okayama University Hospital, Okayama, Japan; 4 Department of Pathology and Oncology, Graduate School of Medicine, Dentistry and Pharmaceutical Sciences, Okayama University, Okayama, Japan; 5 Department of Hematology, Oncology and Respiratory Medicine, Okayama University Graduate School of Medicine, Dentistry and Pharmaceutical Sciences, Okayama, Japan

**Keywords:** Pulmonary sclerosing pneumocytoma, Ipilimumab, Nivolumab, Programmed cell death ligand 1, Case report

## Abstract

Pulmonary sclerosing pneumocytoma (PSP) is a rare form of lung cancer that occasionally presents with lymph node and extrapulmonary metastases, and multiple lesions. The treatment of metastatic PSP remains undefined. This study reports the case of a 48-year-old female patient diagnosed with PSP following surgical intervention for a solitary nodule in the left lower lobe. Four years later, recurrence occurred in the left hilar and mediastinal lymph nodes, necessitating an additional resection. Concurrently, sacral metastases developed and required palliative radiotherapy. Genetic analysis identified an *AKT1* E17K mutation, characteristic of PSP, and absence of programmed cell death ligand 1 (PD-L1) expression in the tumor. Two years post-recurrence, the tumor recurred in the left mammary gland and mediastinal lymph nodes. Combination immunotherapy with ipilimumab and nivolumab yielded a significantly positive response in this metastatic PSP case. This is the first reported case of successful treatment of multiple distant metastatic PSP with ipilimumab and nivolumab, following the failure of various local treatments. Further case series are warranted to validate the efficacy of immunotherapy in metastatic PSP.

## Introduction

Sclerosing hemangioma, a tumor now classified as pulmonary sclerosing pneumocytoma (PSP) under the 2015 WHO classification of lung tumors [1], is a rare occurrence comprising 1–5% of primary lung tumors [2]. Predominantly affecting middle-aged women, lymph node metastasis occurs in approximately 2–4% of cases [3, 4]. PSPs with malignant behavior rarely exhibit distant metastasis [1]. Owing to their relatively benign nature and low prevalence, surgical resection including enucleation, lobectomy, or partial resection, remains the only curative treatment [6]. Currently, metastatic cases lack a definitive treatment approach. This report presents a case of metastatic PSP effectively managed with ipilimumab and nivolumab.

## Case presentation

**Fig. 1:**
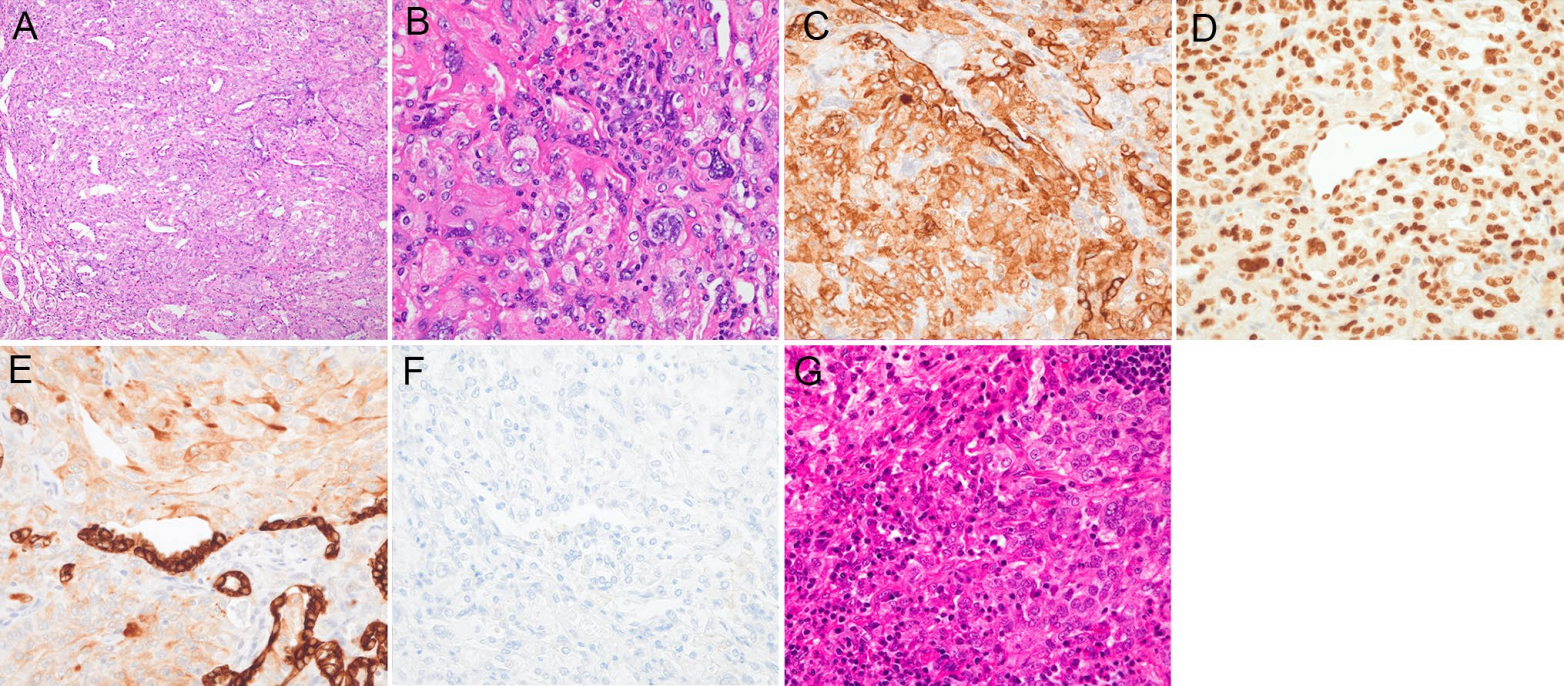
**A–F** Pathological findings of a nodule in the left lower lobe excised during thoracoscopic surgery and diagnosed as a PSP. **G** Pathological findings of dissected mediastinal and lower paratracheal lymph nodes during thoracotomy confirming PSP metastasis. **A**, **B**, and **G** Hematoxylin and eosin staining. **C**–**F** Immunostaining for EMA, TTF-1, CK AE1/AE3, and PD-L1. **A**, **B** Atypical round stromal cells with increased proliferation and pleomorphism compared to that of normal tissues. The surface and round cells are positive for (*C*) EMA and (*D*) TTF-1. The former exhibits diffuse positivity, and the latter is weakly positive for (*E*) CK AE1/AE3, while both are negative for (**F**) PD-L1. Except for image **A**, which was captured with a 100 × objective, all images were obtained using a 400 × objective lens. *PSP* pulmonary sclerosing pneumocytoma, *EMA* epithelial membrane antigen, *TTF-1* thyroid transcription factor-1, *PD-L1* programmed death-ligand 1; *CK* creatinine kinase

A 48-year-old female with a 21 pack-year smoking history and no significant medical history presented with abnormal lung shadows during medical examination and underwent video-assisted thoracoscopic surgery for a nodule located in the left lower lobe. The pathological analysis led to a diagnosis of PSP (Fig. 1A, B), as demonstrated by positive immunostaining for epithelial membrane antigen (EMA), thyroid transcription factor-1 (TTF-1), and cytokeratin (CK) AE1/AE3 (Fig. 1C–E), with prominent round stromal cell proliferation and pleomorphism compared to normal tissue. Four years post-diagnosis, computed tomography (CT) revealed enlarged left hilar lymph nodes, and positron emission tomography-computed tomography (PET-CT) showed fluorodeoxyglucose (FDG) uptake in the left hilar, mediastinal lymph nodes, and sacrum. Transbronchial needle aspiration (TBNA) of the mediastinal lymph nodes confirmed PSP recurrence. Thoracotomy with mediastinal and lower paratracheal lymph node dissection, followed by pathological examination, confirmed metastasis of PSP (Fig. 1G) with immunostaining patterns identical to those of the dissected nodule in the left lower lobe. Stereotactic body radiotherapy using 35 Gy in five fractions was administered to the remaining sacral metastases. The patient was monitored without further treatment and no signs of recurrence were observed.

**Fig. 2:**
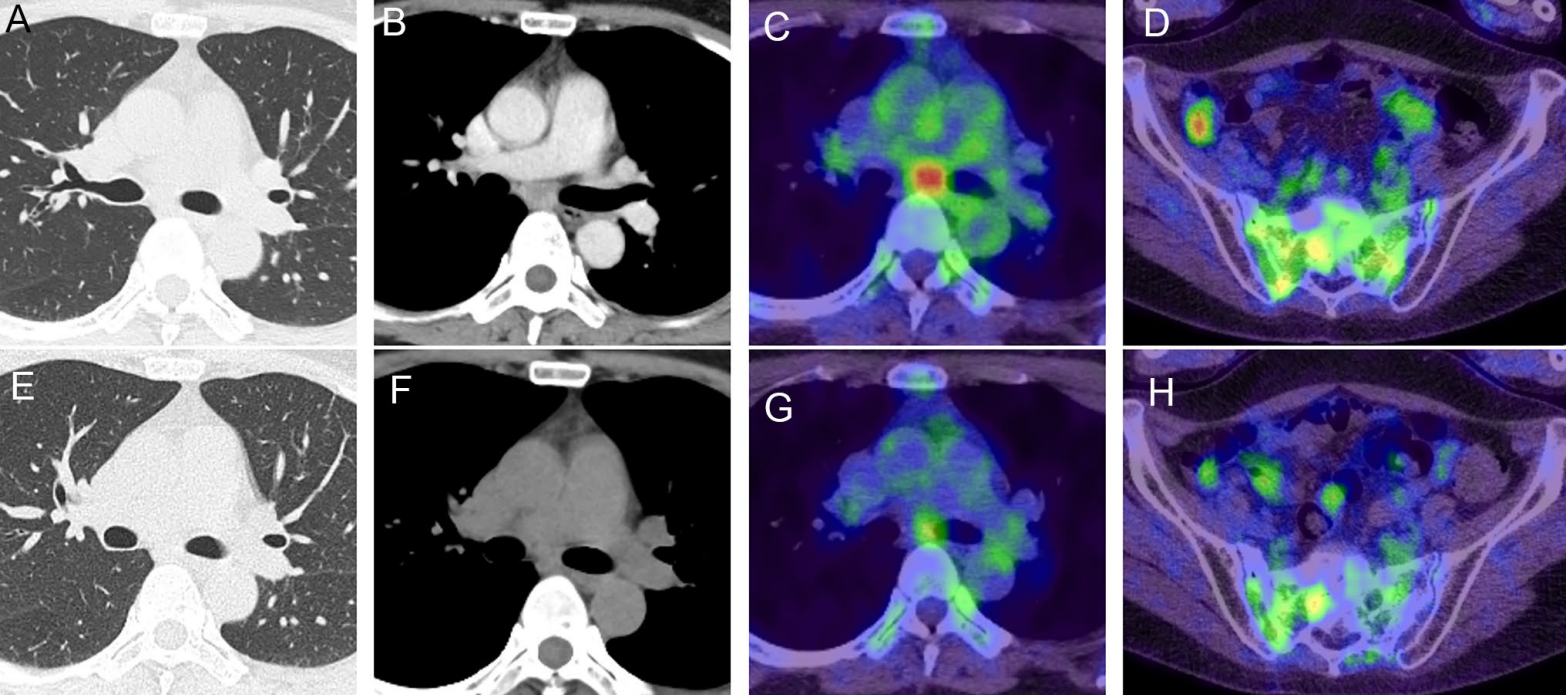
**A**, **B**, **E**, and **F** Chest CT. **C, D, G, H** PET-CT. **A**–**D** reveal enlarged mediastinal lymph nodes and FDG uptake in the lymph nodes and sacrum, indicating recurrent PSP. **E**–**F** Images obtained after three immunotherapy cycles revealing reduced mediastinal lymph node size and FDG accumulation. *CT* computed tomography, *PET-CT*, positron emission tomography-computed tomography, *FDG* fluorodeoxyglucose, *PSP*, pulmonary sclerosing pneumocytoma

Two years after the first recurrence in the lymph nodes and sacrum, a mass was detected in the left breast during breast cancer screening. Fine-needle aspiration biopsy identified this as a metastasis of PSP. PET-CT revealed renewed accumulation in the mediastinal lymph nodes, indicating PSP recurrence (Fig. 2A–D). FoundationOne CDx multigene panel test identified *AKT1* E17K, a common mutation in PSP [7], and two TP53 mutations (R280T and E287K) with a low tumor mutation burden (3 muts/Mb). The tumor proportion score for programmed cell death ligand 1 (PD-L1) expression was less than 1%, as assessed using the 22C3 antibody (Fig. 1F). Consequently, the patient opted for systemic drug therapy over local treatments such as surgery and radiotherapy. Given that the effectiveness of cytotoxic anticancer drugs is typically poor in patients with PSP, we selected combination therapy with nivolumab and ipilimumab (1 mg/kg of ipilimumab every 6 weeks and 360 mg of nivolumab every 3 weeks) upon consensus of multiple oncologists at our institution. This treatment regimen produced no significant adverse reactions and after three cycles, CT and PET-CT revealed a reduction in mediastinal lymph node size and FDG accumulation (Fig. 2E–H). The patient continued to receive ipilimumab and nivolumab therapy for 5 months.

## Discussion

PSP, a type of lung tumor, exhibits a low lymph node metastasis rate of 2–4% [4], with distant metastases being exceedingly rare. Metastatic PSP is rare and few case reports have documented distant metastases [5, 8]. This case report documents the first PSP case with multiple distant metastases that responded to treatment with ipilimumab and nivolumab following the failure of repeated local treatments.

In this case, the PSP was TTF-1 positive, indicating its origin from type 2 alveolar epithelial cells [3]. Thoracic tumors, including non-small cell lung cancer (NSCLC), generally respond well to immunotherapy; however, the efficacy of immune checkpoint inhibitors in patients with PSP has rarely been reported. A previous study demonstrated the efficacy of pembrolizumab in metastatic PSP, although it was not evaluated in combination with apatinib and PD-L1 [8]. The 5-year CheckMate227 Part 1 results indicated that ipilimumab and nivolumab improved overall survival (OS) compared to chemotherapy in patients with metastatic NSCLC, particularly those without PD-L1 expression in the tumor [9]. Hence, ipilimumab and nivolumab were chosen to treat this metastatic PSP case based on the absence of PD-L1 expression.

The AKT signaling pathway is critical for tumor growth, particularly the *AKT1* E17K mutation, which is found in 1.2% of all cancers. The NCI-MATCH study indicated that capivasertib, an AKT inhibitor, may be effective for E17K-mutated tumors with a 28.6% response rate [7]. *AKT1* E17K has also been reported in PSP [8], as demonstrated in this case. However, another study suggested that the *AKT1* E17K mutation alone does not exhibit malignant behavior and that combining *AKT1* mutations with other oncogenes could accelerate the malignant progression of benign PSPs [8]. Thus, the targeted inhibition of AKT alone may be insufficient.

This study has certain limitations. As PSP is a low-grade tumor, it is questionable whether a 7-month treatment duration is sufficient to assess the efficacy of ipilimumab and nivolumab. Additionally, the small size of the mammary biopsy sample precluded genomic or PD-L1 evaluation. Despite these limitations, our findings hold significance for oncologists in similar scenarios.

## Conclusions

This case report describes a patient with metastatic PSP and *AKT1* E17K mutation without PD-L1 expression in the tumor. The patient responded significantly to ipilimumab and nivolumab. Further cases are needed to validate the efficacy of immunotherapy in the treatment of metastatic PSP.

## Abbreviations

CK: Cytokeratin
CT: Computed tomography
EMA: Epithelial membrane antigen
FDG: Fluorodeoxyglucose
NSCLC: Non-small-cell lung carcinoma
OS: Overall survival
PD-L1: Programmed cell death ligand 1
PET-CT: Positron emission tomography-computed tomography
PSP: Pulmonary sclerosing pneumocytoma
TBNA: Transbronchial needle aspiration
TTF-1: Thyroid transcription factor-1
